# Improving the Health and Well‐Being of Adults With Conditions of a Genetic Origin: *Views from Professionals, Syndrome Support Groups and Parents*

**DOI:** 10.1111/jar.12293

**Published:** 2016-10-24

**Authors:** Marcus Redley, Merel Pannebakker, Anthony Holland

**Affiliations:** ^1^ Cambridge Intellectual and Developmental Disabilities Research Group University of Cambridge Cambridge UK; ^2^ Department of Public Health and Primary Care University of Cambridge Cambridge UK; ^3^ National Institute for health Research (NIHR) Collaboration for Leadership in Applied Health Research and Care (CLAHRC) East of England at Cambridge and Peterborough NHS Foundation Trust Cambridge UK

**Keywords:** genetics, health inequalities, healthcare, neurodevelopment syndromes, social care, intellectual disability

## Abstract

**Background:**

Advances in medical genetics herald the possibility that health and social care services could be more responsive to the needs arising from a person's genotype. This development may be particularly important for those men and women whose learning disability (known internationally as intellectual disability) is linked to a neurodevelopmental condition of genetic origin.

**Method:**

This possibility is tested through interviews with samples of (i) professional ‘opinion former’ with nationally recognised clinical and/or academic interests in learning disabilities and genetics; (ii) representatives of syndrome organisations prompting the interests of families where someone has a neurodevelopmental condition, and parent‐members of these same organisations.

**Results:**

The reporting and discussion of the interview data considers the possibility that notwithstanding the successes of the social model of disability, the health and wellbeing of people whose learning disability is associated with a neurodevelopmental condition could be better served by a more medicalised approach to their interests.

**Conclusion:**

While a more medicalised approach to this populations’ disabilities would appear to be beneficial, so long as it is focused on interventions to improve their lives rather than catalogues their deficiencies.

## Introduction

Medical genetics, which relates genetic variation to the burden of disease and ill health, heralds both individualized medicine (Evans & Relling 2004) and the prospect of genetically informed public health policy (Stewart 2007). However, these revolutionary possibilities seem to be just that – possibilities (Lander 2011). Identifying the gene(s) responsible for many common health conditions (such as schizophrenia) and then targeting these risks at the individual or population level is proving difficult because many common illnesses are a result of complex interactions between multiple genes, as well as a person's environment (Ripke *et al*. 2014). Yet for a range of conditions associated with either a chromosomal abnormality, or a mutation in a single gene, this is not the case. These genetic conditions, often associated with significant learning disabilities (known internationally as intellectual disabilities), and frequently referred to as neurodevelopmental disorders or syndromes, are already associated with well‐documented patterns of ill health and also particular developmental profiles and propensities to specific problem behaviours (see O'Brien 2006). Down's syndrome, for instance, the most common and familiar neurodevelopmental syndrome, is due to the inheritance of an extra copy of chromosome 21 (trisomy 21). Men and women with Down's syndrome are at increased risk of a number of illnesses and symptoms, including congenital heart disease, reduced physical activity, weight gain, depression, sensory impairments and early onset dementia (http://www.nhs.uk/conditions/downs-syndrome/pages/introduction.aspx). What is striking is that these health risks, and those associated with many other neurodevelopmental disorders of genetic origin, have very little influence on the design and delivery of health care (Department of Health, 2013). In other words, while members of the general population are poised to benefit from advances in medical genetics, people with neurodevelopmental disorders of a genetic origin – whose health risks are much better documented – are not. This could be seen as yet more evidence that the UK's healthcare system is failing to meet the needs of people with learning disabilities (Heslop *et al*. 2014). Aiming to investigate this possibility, we consider whether a more judicious use of genetic knowledge in the design and delivery of healthcare and social care services could improve the health and well‐being of those people whose learning disability has a genetic cause. A provocative question as it has the potential to reignite past controversies concerning the eugenics movement of the late 19th and early 20th centuries, such as illustrated by the reported case of the Kallikak Family (Goddard 1912), while also raising the possibility that these populations might benefit from a re‐medicalization of their learning disabilities, despite the orthodoxies of the social model (Barnes 2012). Prior to exploring these possibilities through interviews with key stakeholders, we briefly introduce how the association between neurodevelopmental disorders that have a genetic origin and particular pattern of ill health is understood; review the current use of genetic knowledge in the provision of health and social care services; and highlight the emergence of syndrome‐specific support groups representing the interests of families where a member has a genetic neurodevelopmental condition. We then report on original research in which key stakeholders gave their views on the potential of medical genetics to improve the health and well‐being of these populations. Views that are then used to discuss whether, and how, a more judicious use of genetic knowledge might improve the health and well‐being of people whose learning disability is associated with a genetic condition.

## Background

Conditions of genetic origin have a characteristic and identifiable genotype that in many cases provides a definitive diagnosis when a genetic disorder is suspected. In some instances, these genetic conditions will be inherited, carried by a parent (perhaps in a milder form) and passed down to a proportion of their children. This is the case with tuberous sclerosis, which is due to a mutation in either the TSC1 or TSC2 gene, or, as in the case of fragile X syndrome, a mutation in the FMR‐1 gene that is passed from a mother to her son who is likely to be more severely affected due to the mode of inheritance (referred to as X‐linked). In other instances, a genetic condition may be due to a new (*de novo*) event in either the sperm or ovum, giving rise to a genetic ‘abnormality’ (mutation); or to the loss or duplication of a few genes on a particular chromosome (referred to as a copy number variant); or, as is the case in most people with Down's syndrome, the inheritance of an extra copy of a whole chromosome. In Prader–Willi syndrome, for example the genotype is characterized by the absence (or non‐expression) of what are referred to as ‘imprinted’ genes located on part of chromosome 15. Where a genetic condition is due to a *de novo* event, the chances of a recurrence in that generation are low, and it is also unlikely that those with such neurodevelopmental syndromes will, themselves, go on to have children. It is also the case that many of the embryos and the subsequent foetuses affected by these *de novo* events will not survive gestation.

The consequence of a person's genotype is evident (expressed) in both the form of a physical and also a behavioural phenotype, the phenotype being the characteristics associated with that particular genotype (see Table 1). With respect to a person's physical phenotype, this can include distinctive facial features (associated with many neurodevelopmental syndromes), characteristic physical and sensory disabilities and, of particular concern here, specific patterns of ill health. For example, people with Cornelia de Lange syndrome exhibit, in addition to a set of characteristic facial features and low birth weight, a propensity to a sideways curvature of the spine (known as scoliosis) and to gastroesophageal reflux in which acid from the stomach enters and burns the oesophagus causing extreme pain. In addition to a distinct array of physical features, researchers have also identified that many of these disorders of genetic origin are associated with particular styles of cognitive processing, particular behavioural characteristics and styles of social interaction, some of which may meet criteria for autism spectrum disorder (O'Brien 2002). Known as a ‘behavioural phenotype’, these consequences of a person's genotype can therefore include, amongst others, a learning disability, autism, repetitive behaviours, social disinhibited behaviours, abnormal eating behaviours and high rates of obesity, and self‐injurious behaviour. In addition, some people may also be at high risk of anxiety disorders and/or psychotic illnesses. For example, people with velocardiofacial syndrome (VCFS) may, in addition to a learning disability, have the developmental profile characteristic of autism and be at risk for developing schizophrenia. While some commentators choose to see psychiatric diagnoses such as schizophrenia, as social constructions (Rapley 2004), others recognize their utility in enabling treatment developments, such as medications, that, as part of a comprehensive treatment programme, have been shown to have some benefit and to improve the well‐being of those affected (see Miyamoto *et al*. 2012). Before knowledge of a person's genetic condition – its genotype and physical and behavioural phenotypes – can be used to improve a person's health, it is necessary to develop interventions using that knowledge.

**Table 1:** Syndromes and some of the features associated with their physical and behavioural phenotypes^a^

Syndrome	Features
Angelman	Genotype: Chromosome 15 (10% unknown genetic cause) *de novo* mutation; physical phenotype: gait ataxia, distinct facial features, epileptic seizures; behavioural phenotype: intellectual disability, speech impairment, inappropriate demeanour including frequent laughter and excitability
Cri du Chat	Genotype: Chromosome 5; Mostly *de novo* mutation, 10% inherited; physical phenotype: cat like cry, distinct facial features low birth weight and slow growth, scoliosis gastroesophageal reflux; behavioural phenotype: intellectual disability, slow or incomplete development of motor skills
Cornelia de Lange	Genotype: Chromosome 5, 10 or X (35% unknown genetic cause); *de novo* mutation; physical phenotype: restricted growth, distinct facial features, gastrointestinal dysfunction, hearing loss; behavioural phenotype: intellectual disability, autism, self‐injury
Down's	Genotype: Chromosome 21 (trisomy 21); *de novo* occurrence; physical phenotype: poor muscle tone, distinct facial features, slow physical development, heart problems as well as hearing and eye problems, underactive thyroid; behavioural phenotype: intellectual disability, early onset dementia
Fragile X	Genotype: X chromosome; inherited (if mother is carrier) and *de novo* mutation; physical phenotype: distinct facial features, laxity of joints, heart problems; behavioural phenotype: moderate intellectual disability, mild intellectual disability in females, autism
Prader–Willi	Genotype: Chromosome 15; *de novo* chromosomal abnormality; physical phenotype: hypotonia and feeding difficulties in early infancy, excessive eating and morbid obesity in adulthood, distinct facial features short stature, strabismus, scoliosis; behavioural phenotype: intellectual disability, delayed language development, temper tantrums, psychosis
Rett	Genotype: X chromosome; mostly a *de novo* mutation; physical phenotype: presumed to be lethal in males, females, developmental stagnation, regression in language and motor skills, episodic apnoea and/or hyperpnoea, gait ataxia and apraxia, tremor, epileptic seizures, distinctive facial features, restricted growth, constipation, scoliosis; behavioural phenotype: autistic features, intellectual disability, repetitive and stereotypic behaviour, panic‐like attacks, episodes of inconsolable crying and screaming
Rubinstein–Taybi	Genotype: Chromosome 16 (40–60% unknown genetic cause); *de novo* mutation; physical phenotype: distinctive facial features, short stature, propensity towards obesity, eye problems, congenital heart conditions, renal abnormalities; behavioural phenotype: intellectual disability, intolerance of noise and crowds, autism, hyperactivity, self‐injury, aggression
Tuberous sclerosis	Genotype: Chromosome 9 or 16 (15% unknown genetic cause); *de novo* mutation, 30% inherited; physical phenotype: benign tumours causing problems in the kidneys, heart, lungs and brain; behavioural phenotype: intellectual disability
Williams	Genotype: Chromosome 7; *de novo* chromosomal abnormality; physical phenotype: cardiovascular disease, distinctive facial features, abnormalities in connective tissue the endocrine system and in growth; behavioural phenotype: intellectual disability, over friendliness and social disinhibition
22q11 deletion	Genotype: Chromosome 22; *de novo* chromosomal abnormality and 7% inherited; physical phenotype: congenital heart disease, abnormalities in the palate, immune deficiencies, distinctive facial features; behavioural phenotype: intellectual disability, autism, high incidence of schizophrenia

a The information presented here was gathered from Genetics Home Reference (ghr.nlm.nih.gov) a service of the National Library of Medicine (NLM) and part of the National Institutes of Health: an agency of the U.S. Department of Health and Human Services.

In the biomedical sciences, researchers have demonstrated an ability to switch off the excess gene expression associated with trisomy 21 in Down's syndrome, albeit in isolated cells (Sample 2013): a discovery which raises the possibility that at some point in future Down's syndrome might be ‘treatable’ *in utero*. No less dramatic is the use of mouse models of specific genetic syndromes to demonstrate the possibility of ‘switching on’ the MECP2 gene, which is ‘switched off’ in Rett syndrome (Guy *et al*. 2007); another piece of research that hints at future ‘cures’. Of possibly more immediate benefit is the development of pharmaceuticals that have the potential to shrink the tumours associated with tuberous sclerosis (Franz *et al*. 2006). With respect to studies of behavioural, as opposed to the physical phenotypes, research suggests that what appear to be ostensibly similar behaviours – self‐injury, temper tantrums and aggression – might have entirely different causes (Oliver *et al*. 2013). The implication here is that, while awareness of a person's syndrome does not fully explain his or her behaviour, it may help to develop interventions targeted at the causal pathways that lead to those behaviours. A possibility that would lessen the need to rely upon generic interventions for something so poorly defined as ‘challenging behaviour’ (Woodcock *et al*. 2009). This research also has implications for social care: care managers who are more aware of a person's behavioural phenotype will be more mindful of the demands falling on family carers and thus their capacity to cope (Adams *et al*. 2012).

Successive governments have done much to address the fact that people with disabilities and rare health conditions can be disadvantaged in a healthcare system designed around the needs of the majority population (Heyman, Swain, and Gillman 2004). For instance, the *Autism Act 2009*, the first and only disability‐specific legislation in England and Wales, requires local authorities and NHS organizations to develop services that support and meet the needs of those affected by this condition. Similarly, the *Equality Act 2010,* should ensure that the needs of all people with disabilities are accommodated when receiving health and/or social care services. Nonetheless, there are consequences of having a genetic condition that are not so readily remedied by antidiscrimination legislation. Namely, the sheer rarity of these conditions mitigates against the possibility of establishing robust evidence bases when evaluating new or alternative courses of treatment. In recognition of this, the government developed a *Strategy for Rare Diseases* (Department of Health, 2013) covering disorders/illnesses that affect five or fewer persons per 10 000 of the population. The strategy has five main elements that include the following: involving those affected in implementing the strategy; timely diagnoses and early intervention for known health problems; prenatal screening so that prospective parents can make informed choices when deciding to start a family; the provision of coordinated and multidisciplinary care; and researching the most effective approaches when caring for people affected by a rare disease. The strategy is unfunded and given that it takes, on average, 17 years before biomedical research begins to affect clinical practice, and (Cooksey 2006) it will be a while before the success (or otherwise) of this strategy can be assessed. In sharp contrast to this top‐down strategy, there are also a growing number of support groups representing the interests of families where one or more members are affected by a rare disease. With respect to those support groups that specifically represent the interests of people with neurodevelopmental conditions of genetic origin, these began forming in the early 70s – see Table 2 – with the founding of the Down's Syndrome Association. Since then their numbers have grown, partially as a consequence of the knowledge arising from the Human Genome Project (1990–2003) and the increasing availability of genetic tests (Peters *et al*. 2015). Sometimes characterized as ‘embodied health movements’ (Brown, Zavestocki *et al*. 2004), while some of these groups seek alternative therapies and treatments, others actively embrace the medicalization of their condition. Moreover, with the aim of hastening the development of cures and new treatments, these groups tend to be very supportive of, and willing to participate in, medical research (Koay & Sharp 2013). It has even been suggested that identities formed around genetic diagnoses could replace more traditional loyalties based on class and ethnicity (Rose & Novas 2004). Biological citizenship, grounded in genetic identities and committed to medical research, poses a direct challenge to the Disabled People's Movement with its commitment to the social model and the view that *disability*, irrespective of diagnostic specifics, is rooted in a shared experience of discrimination and oppression. Indeed so divergent are these two perspectives that disability activism, despite the very obvious political success of the social model (Shakespeare 2005), could be bifurcating along these lines (Hughes 2009).

**Table 2:** The 11 syndrome support groups involved in the study

Syndrome support group	Foundation year	Number of member households^a^	Prevalence of syndrome per 250 000 of the population^b^	Estimation of UK population affected by the syndrome	Percentage of affected population who are syndrome group members
Angelman Syndrome Support Education & Research Trust	1993	300–350	6	1600	21
Cri du Chat	1989	80	5	1300	6
The Cornelia de Lange Foundation	1988	360–450	5	1300	32
Down's Syndrome Association	1970	20 000	313	79 000	25
The Fragile X Society	1990	1850	100	25 300	7
Prader–Willi Syndrome Association UK	1982	800	5	1200	66
Rett UK	1985	695	25	6300	11
Rubinstein–Taybi Syndrome UK Support Group	1986	173	2	500	34
Tuberous Sclerosis Association	1977	727	43	10 900	7
Williams Syndrome Foundation	1980	1000	13	3200	32
Max Appeal! (22q11.2 deletion syndrome support group)	2000	425	63	15 800	3
Prevalence of all eleven syndromes and estimate of UK population affected	–	–	479	146 563	–

a The membership records of some of these organizations were not always up to date.

b Population of UK in 2012 was 63.32 million rounded up to the nearest hundred.

It is in the context of research that clearly associates neurodevelopmental disorders of a genetic origin with particular patterns of ill health, an awareness of discrimination in the provision of health care and potential disadvantage in the development of new treatments, and a grass roots mobilization around genetics diagnoses, that we are asking: could a more judicious use of genetic knowledge in the design and delivery of healthcare and social care services improve the health and well‐being of those people whose learning disability has a genetic cause? A question that we sought to address by canvassing the views of selected stakeholders.

## Stakeholder views on the utility of syndrome‐specific knowledge

The stakeholders we interviewed comprised a sample of: (i) ten persons we are referring to as ‘professionals’ who the authors recognized as having both an interest in learning disabilities and genetics, and national, if not international, reputations in this area; (ii) representatives from eleven syndrome organizations that promote the interests of those affected by neurodevelopmental conditions of a genetic origin, and these responders were identified through their involvement in a small project funded under the Medical Research Council's (MRC) Lifelong Health and Wellbeing initiative; and (iii) fifty‐five parent members, five from each of the eleven organizations described in Table 2. The syndrome organization representatives identified these parent members. The interviews were semi‐structured, the major themes addressed in the interviews being determined by our research interests and the fact that the experiences and expertise of these three groups of respondents are significantly different. The interviews with the ‘professionals’ addressed the practical utility of using syndrome‐specific knowledge to improve the healthcare and social care services received by people whose neurodevelopmental syndrome has a genetic origin; the representatives from the syndrome organizations were asked about the aims of their respective organizations and their efforts to promote the interests of their members; while the interviews with the parent members of these organizations focused on their experiences of raising a child, and in some cases, a now adult child with a neurodevelopmental condition. However, in order to develop a lively discussion where respondents could develop their own lines of thinking and reasoning, the interviewer (Merel Pannebakker, the second author) was encouraged to adopt a lively conversational style (Holstein & Gubrium 1997). All the interviews, in order to keep costs to a minimum, were conducted over the telephone and answers were recorded contemporaneously by hand, with only key phrases or expressions preserved verbatim. The interviews, which lasted between 45 and 90 min, were then examined for content (Cirourel 1964), with emergent themes identified and coded. As it was our intention only to document arguments for‐and‐against a more judicious use of genetic knowledge in the design and delivery of health and social care (not analyse respondents’ construction of their subjective experiences), no quotations from the interview data are presented. Ethical approval for the project was sought from NRES (www.nres.nhs.uk/) who classified the project as a service evaluation. The views of the ‘professionals’ are reported first, followed by those of the representatives of the syndrome organization, and finally, those of the parent members of these organizations.

### Professionals

The sample of ten professionals comprised three persons with an in‐depth knowledge of one or more neurodevelopmental syndromes associated with learning disabilities (two psychiatrists and a psychologist) and six generalists with a clinical and/or academic interest in the genetic basis of learning disabilities but lacking specialist knowledge of any one neurodevelopmental syndrome (two clinical geneticists, a psychiatrist and one each of the following: a psychologist, a nurse with learning disability and a disability studies scholar).

All but one of the ‘professionals’ endorsed the idea that knowledge of a person's neurodevelopmental syndrome – over and above any general understanding of his or her learning disability – could, and should, play a decisive role in the provision of health care. This view was based on the belief that with knowledge of a patient's neurodevelopmental syndrome, and in particular its physical phenotype, healthcare practitioners could actively look for symptoms of syndrome‐related illnesses, rather than merely respond to signs of ill health. Adopting such an approach, it was asserted, would be especially beneficial where patients, because of communication difficulties, were unable to give reliable reports of their symptoms. In a similar vein, a number of respondents suggested that annual health checks, which many people with a learning disability are entitled to (Michael 2008), should be specially adapted so as to ensure they include those health risks that are related to the person's genetic syndrome. One respondent went further, suggesting that family carers and direct support staff (DSS) proactively make regular appointments with their general practitioners (GPs) for the purpose of reviewing syndrome‐related health risks, rather than waiting until the person concerned showed signs of ill health. That said, these ‘opinion informers’ were well aware that many GPs, as well as healthcare practitioners in secondary services, can be reluctant to engage with patients’ genetic conditions. A claim that was substantiated by observing that when parents proffered information sheets concerning a son or daughter's condition, these were routinely ignored. Although critical of this, these professionals understood why this might be occurring, suggesting, for example that clinicians may have doubts about the validity of the information provided or that a patient's genetic syndrome may not be relevant to the health condition being treated. When asked how best to improve clinicians’ knowledge of patients’ genetic syndromes, many of these respondents had little to suggest other than more or better training and education. This suggestion often came with a caveat that, as many of these syndromes are extremely rare, a clinician may never meet a patient with one of these neurodevelopmental disorders. A more imaginative proposal was that clinicians received training in the principles of caring for patients with rare genetic conditions. The fact that there are variations in the health needs of people with the same neurodevelopmental condition was not seen by these respondents as undermining the perceived clinical value of syndrome‐specific knowledge except, that is for the two respondents, otherwise committed to the clinical utility of knowing a person's physical phenotype, who questioned the clinical benefits of knowing a person's behavioural phenotype. These two respondents were of the view that behavioural phenotypes lacked specificity as the same behaviours can occur in more than one syndrome (Hodapp & Dykens 2004). Only one ‘professional’ raised the prospect of syndrome‐specific medication, highlighting the treatment of brain tumours in tuberous sclerosis (Franz *et al*. 2006), a breakthrough that this respondent saw as likely to extend people's lives and dramatically improve their well‐being. That only one respondent drew attention to the advance in syndrome‐specific medication may reflect our small sample size but also it may signal the belief that neurodevelopmental conditions are essentially incurable.

The one ‘professional’ who was critical of health care adopting a syndrome‐specific focus held the view that improving health care for these patients depended upon improving health care of all patients with learning disabilities, not just those with a genetic syndrome. In the opinion of this respondent, knowledge of patients’ neurodevelopmental conditions could never play a decisive role in the provision of health care because the rarity of these syndromes precluded the development and implementation of the necessary clinical expertise. Far more important for this respondent – who acknowledged the existence of syndrome‐specific patterns of ill health – was ensuring that patients received person‐centred care and treatment. This, it was believed, would ensure that people with a neurodevelopmental condition received health care that was appropriate to both their syndrome‐specific health conditions as well as those health problems they might share with the general population, including people whose learning disability does not have a genetic origin. Amongst those respondents who otherwise advocated a more syndrome‐specific focus to health care, some shared concerns similar to those of this respondent. These respondents mentioned, for instance, that for at least a third of all people with a learning disability, there was no identifiable cause (genetic or otherwise); that an emphasis on people's physical and behavioural phenotypes might lead to them being seen as ‘ticking time‐bombs of ill health’; and that developments in prenatal screening might lead to an unwelcome increase in the number of pregnancies being terminated.

Commenting on the utility of syndrome‐specific knowledge in the provision of social care services, two respondents thought it had no place. In their view, social care was primarily concerned with daily life and community participation and, as such, it should be person centred. Focusing on people's medical conditions, they thought, would lead to a kind of ‘genetic fatalism’ thereby lowering expectations of what a person could achieve. In sharp contrast, the other eight respondents emphatically believed that providers of social care should take far more interest in people's genetic conditions. This opinion rested not just on the increasing integration of health and social care (Department of Health, 2011), but on the fact that a significant amount of health care – identifying symptoms and following treatment plans – already occurs within social care settings. In the judgement of these respondents, good health care depends on direct support staff being aware of a client's syndrome‐specific health risks. One of these respondents was of the opinion that it was time to acknowledge that people labelled as having a ‘learning disability’ comprise a diverse population and that being person centred means being aware of a person's genetic syndrome and his or her syndrome‐specific health risks. Allied to this, another respondent observed that the social care needs of family carers also vary significantly depending on the neurodevelopmental condition affecting a son or daughter, a point that was illustrated by the story of a child with Cornelia de Lange syndrome whose parents became the subjects of a safeguarding inquiry. The boy's care manager failed to appreciate that signs of malnourishment and severe bruising could be a consequence of chronic reflux and self‐injurious behaviour both features of the physical and behavioural phenotype for people with Cornelia de Lange syndrome. This same respondent, a strong advocate for the relevance of behavioural phenotypes in social care, also suggested that when social service departments are promoting a person's social inclusion they often overlook syndrome‐specific risk. For example, for people with Angelman syndrome, the possibility of forming inappropriate relationships due to a desire for social attention (Oliver *et al*. 2013); in Prader–Willi syndrome, the risk of overeating and life‐threatening obesity (Holland *et al*. 2003); and in the case of people with autism, which is associated with some neurodevelopmental syndromes, an aversion to social encounters.

To sum up: the majority of ‘professionals’ did not foresee breakthroughs in biomedical research as the main route to improvements in the health and well‐being of these populations. Rather, they saw institutional reforms – better training for clinicians in the principles of treating people with rare syndromes, the inclusion of syndrome‐specific health risks in annual health checks and ensuring that direct support staff and care managers are aware of syndrome‐specific health risks – as the factors most likely lead to improvements in people's lives. In other words, these respondents saw the medicalization of people's learning disability – knowing their genotypes and associated physical and behavioural phenotypes – as crucial to improving their health and well‐being. A minority held a contrary view emphasizing instead the importance of adopting a person‐centred approach to meeting people's healthcare needs and they were concerned that, by defining people by their syndrome‐specific disabilities and health problems, this would lead inevitably to genetic fatalism and pessimism.

### Syndrome organizations’ representatives

The respondents representing the eleven syndrome organizations were, in all but one case, parents of a child with a neurodevelopmental condition. Interestingly, these organizations counted their membership in terms of households – one or more parents and a child with the ‘qualifying’ syndrome – rather than in terms of individuals. Unsurprisingly, the representatives saw their respective memberships as different from the wider population of people with a learning disability. These differences they described in terms of phenotypical differences in facial features, physical and sensory disabilities, and known health risks. However, these respondents were also keen to stress the heterogeneity of their memberships. They noted, for instance, variations in the severity of people's learning disabilities, the degree to which people could be affected by comorbid physical and sensory impairments and differences in which people with the same syndrome were able to participate in society. Nonetheless, having the same genetic identity is the defining characteristic of those in these support groups. The significance of this identity, moreover, was apparent in the kinds of organizations these support groups chose to be affiliated with: only two of the eleven representatives reported affiliations with national UK learning disability charities, while all eleven representatives described affiliations to organizations representing people with specific medical conditions (such as epilepsy), rare diseases and umbrella groups for people with genetic conditions. Moreover, many representatives reported links to similar syndrome‐specific groups in other anglophone countries.

When asked about the aims of their organizations, all the representatives answered in broadly similar terms mentioning the offering of advice and information to families, providing social opportunities so that these families could meet and encourage each other and supporting biomedical research that might benefit their memberships. Of these activities, providing parents with information concerning a child's genetic condition was seen as particularly important. This information provided parents with some indication of the life they and their son or daughter was likely to lead, while also equipping parents with such information as might be useful when in conflict with health and social care services. The support these organizations offered to researchers was predominantly assistance with recruiting research participants and such research came through close ties to individual academics. These academics would often sit on support group's scientific advisory panels and speak at annual meetings on the latest research findings.

With respect to the provision of health care, these representatives were, in the main, critical of all healthcare practitioners except paediatricians, who were seen as better informed and more willing to engage with a child's genetic condition. In some cases, this criticism of healthcare practitioners was tempered by an awareness that these clinicians, because of the rarity of the syndrome, did not regularly meet patients with these conditions. Nonetheless, parents were described as extremely distressed when a healthcare practitioner displayed a reluctance to learn about their son or daughter's genetic condition. As a means of addressing this problem, two of the syndrome organization representatives proposed the introduction of syndrome‐specific clinics. These clinics, they envisaged, would be multidisciplinary, convene two or three times a year and held in different parts of the country thus enabling parents to access an integrated health service where all of their child's physical and mental health problems could be addressed by clinicians with the relevant expertise. None of the representatives referred to the introduction of annual health checks, let alone the possibility that these might be extended to include those health conditions specific to a person's genetic syndrome. Three respondents did, however, mention the development of pharmacological treatments for some syndrome‐related health conditions, but for two of these respondents, this was thought to be only a remote possibility. As such, these respondents did not anticipate any kind of medical breakthroughs that would lead to dramatic improvements in people's health. Rather, they were of the opinion that improvements in people's health would come about by ensuring clinicians were better informed about people's syndrome‐specific health risks. With respect to social care, these representatives were less clear about the role that genetic knowledge might play, stressing instead the importance of recognizing a person's potential rather than the disabling effects of his or her genetic condition. What most respondents considered important was that services enabled people to participate in society and access their local communities, and as such, these interviewees did not expect direct support staff to be particularly knowledgeable of a person's genetic condition. Where there was criticism, this was specifically aimed at those care managers who sought to promote the autonomy of service users, irrespective of parents’ worries about a child's syndrome‐specific vulnerabilities. These vulnerabilities included the risk of overeating in people with Prader–Willi syndrome, and the social disinhibition, anxieties and social phobias that are associated, respectively, with Angelman syndrome, Williams syndrome and fragile X syndrome. Care managers were also criticized for failing to appreciate just how demanding it could be caring for a son or daughter with a neurodevelopmental disorder. In summary, these syndrome organizations were strongly committed to their members’ genetic identities recognizing, however, that their affected members varied in their physical and behavioural phenotypes. Genetic identities were seen as paramount when accessing health services, but here again, the route for improving people's health and well‐being was thought to lie in institutional reform – clinicians being more willing to engage with people's genetic conditions and the introduction of multidisciplinary clinics – rather than the discovery of a cure or a revolutionary new treatment. When accessing social services, there was less emphasis on the importance of people's genetic identities but there was, nonetheless, criticism of a care manager who failed to appreciate parents’ syndrome‐specific concerns.

### Parent members

The sample of parent members of these syndrome organizations included 44 parents of younger children (up to the age of 18) and 11 parents of adult children (aged 18 years and over). By comparing the views of these two different sets of parents, our interview data reveals how the significance of a son or daughter's genetic condition can change over time. All the parents we spoke to reported extensive contact with clinical services, especially hospital paediatrics, when it became apparent that their newborn child was visibly disabled, ill and/or failing to thrive. Receiving a diagnosis – whether by clinical assessment or by genetic test – was, for these parents, very important. It gave them a credible explanation for their child's health conditions and developmental delays. Moreover, some parents described how receiving this diagnosis reassured them, in some unspecified way, that they were not responsible for their child's condition, even in the case of inherited conditions. Armed with a diagnosis, as well as an awareness of their son's or daughter's health problems, these parents described how they then set about learning all they could about their child's neurodevelopmental condition, including making contact with the relevant syndrome organization. Parents told us that they really valued these syndrome organizations as they provide opportunities via websites, newsletters, social activities and annual meetings to learn more about their child's genetic condition, the realities of parenting such a child and how to get the best from health and social care services. Despite having a highly medicalized view of their son or daughter's plight, these parents also stressed their child's unique personality and that he or she did not necessarily possess all the traits associated with that particular syndrome. Parents were highly critical of healthcare professionals who were unwilling to learn about their son or daughter's genetic condition. However, it was apparent from the interviews with the parents of older children that they were less concerned with their children's health problems (so long as they were not acute) as these were at least familiar and they had had many years’ experience in responding to them. These parents were concerned about the lack of opportunities for their adult sons and daughters to acquire some degree of independence and possibly move out of the family home. The most significant barriers in achieving greater social inclusion and independence, as identified by these parents, were mental health problems. These were seen as severely limiting opportunities to use public transport and secure paid employment. The debilitating effects of mental health problems were keenly felt by those parents who believed that, but for these problems, their adult children could achieve far more. Yet at the same time, these parents were highly critical of those care managers who, as they saw it, underestimated the extent to which a son or daughter's learning disability and/or their mental health problems made greater independence either unrealistic or very risky. These parents, with few exceptions, did not expect care managers to be particularly knowledgeable about a son or daughter's genetic condition, except where a lack of understanding was thought to put their adult child at risk. This was particularly so for the mothers of three older children with either fragile X, Williams or Prader–Willi syndrome. These mothers complained that the care managers, with responsibility for their adult children, had little comprehension of how these syndromes might impact on a person's life.

When asked about the kind of future they saw for their son or daughter, all the parents we spoke to wanted to see a world in which their children were accepted and, despite their disabilities, had opportunities to participate in society. A minority hoped for a medical breakthrough, but these hopes were largely confined to the treatment of specific health conditions, not a global cure for either their syndrome or the learning disability associated with it. The only exceptions to this were the five mothers of daughters with Rett syndrome; they had hopes of a cure following recent reports in the British media of a possible cure (see BBC News, 2007).

In summary: these parents were committed in their belief as to the benefits that may come from medicalization of their children's disabilities. In this respect, receiving a genetic diagnosis had an existential significance for these parents: it is central to their attempts to understanding both their own and their child's predicament and, as such, they expect both clinicians and care managers to take it seriously. However, as their children reach adulthood, when they might be expected to attain independence, the practical implications of that diagnosis declines and these parents become more concerned about the impact that mental health problems could have on opportunities for social inclusion.

## Discussion

This small‐scale study had limitations, most notably, the absence of views from people with a neurodevelopmental syndrome, and the fact that we did not canvas the opinions of parents who had not joined a syndrome support group. Our recruitment of respondents was also somewhat idiosyncratic and could well have biased our findings. In identifying ‘professionals’, we traded upon our own knowledge of those who we thought could offer well‐informed opinions; while the choice of family members interviewed was entirely under the control of the syndrome organization representatives. In addition, the fact that respondents’ answers were recorded by hand means that we will have lost some of the subtlety of their opinions. Nevertheless, the data collected provides a unique set of materials with which to reflect upon whether a more judicious use of genetic knowledge in the design and delivery of healthcare and social care services could improve the health and well‐being of people whose learning disability has a genetic cause.

Given the sample of stakeholders interviewed, it is perhaps not surprising that an overwhelming majority favoured giving genetic knowledge a more prominent role in the provision of services. This point of view, however, has its subtleties. The benefits of genetics research were not thought to lie in the development of new treatments, but in institutional reforms: training in the principles of supporting patients with rare conditions; the inclusion of syndrome‐specific health risks in annual health checks; the introduction of syndrome‐specific multidisciplinary clinics; and ensuring that direct support staff receive training in the syndrome‐specific health risks of the people they support. The last of these reforms draws attention to the fact that a significant proportion of this population's health care is provided in social care settings by direct support staff with little or no clinical training. Complaints that health care practitioners can be unwilling to engage with patient's genetic syndromes may be harder to address, as these complaints signal deficiencies in the attitudes of clinicians, rather than how services are organized and delivered. Nevertheless, if healthcare practitioners were more sensitive to the existential significance that a son or daughter's genetic diagnosis has for these parents, they might be more willing to learn about it as a means for developing and sustaining a clinical relationship with these parents. It is difficult to see how reforms along these lines could be anything but beneficial, and in full accord with the government's aim of improving health care for people with rare diseases (Department of Health, 2013). When considering why the design and delivery of health care is not more informed by the diagnosis of these conditions, we should perhaps reflect on the fact that ‘learning disabilities’, as part of a medical speciality, has lacked a significant institutional base since the closure of the long‐stay hospitals.

Social care is a particular worry for parents with adult children. This is not, in the main, because care managers fail to recognize the syndrome‐specific needs of their children – although this is a concern for some parents – rather, these parents are distressed about the detrimental impact their son or daughter's mental health problems have on opportunities for social inclusion and greater independence. As such, it is revealing to note that in England neither the Government policy paper *Valuing People* nor its subsequent follow‐up, *Valuing People Now* (Department of Health, 2001, 2009), address, in any detail, the support needs of people with mental health and/or behavioural problems, whether they have a neurodevelopmental syndrome or not. It remains to be seen whether studies of behavioural phenotypes will lead to more effective interventions, both pharmacological and non‐pharmacological, that might lessen the incidence of so‐called ‘challenging behaviour’ (Woodcock *et al*. 2009; Oliver *et al*. 2013) and provide those affected with more opportunities to participate in society.

It is widely believed that when people's disabilities are seen as medical problems, this invariably means focusing on their deficits and what they cannot do. This argument is at the core of the social model of disability and its goal of social reform (Oliver 1990). Yet the parents we spoke to actively embraced a medical understanding of their children's predicaments, saw an understanding of their child's genotype and associated phenotype as essential to promoting their interests and had joined support groups in which membership is defined through a genetic identity. These parents, as well as the overwhelming majority of ‘professionals’, saw medicalization as essential to promoting the health and well‐being of these populations. In other words, there is potentially more to a medical diagnosis than the perceived denigration of a person with a disability; it can become a route to a better understanding of what is deemed a medical condition and to campaigning for more appropriate health care. Nonetheless, these parents, and the representatives from the eleven syndrome organizations, also sought to counter the potentially homogenizing effects of a diagnostic label by emphasizing the uniqueness of each child, including how their son or daughter differed from the physical and behavioural phenotypes associated with their chromosomal condition. Foregrounding a genetic identity flies in the face of the *People First Movement* and its campaigning slogan to ‘label jars not people’ (http://www.peoplefirst.org). It is possible, however, that a genetic label, especially at a time when genetic science is at the forefront of public consciousness, provides a more coherent basis for formulating a campaigning identity than the designation ‘learning disability’, which can encompasses a hugely diverse population. It might also be the case that a genetic identity is less stigmatizing, as the extent and nature of a person's disabilities are not directly revealed, only that the person concerned probably has some special needs. Moreover, given the concerns that these parents have for the health of their children when newborn, it is entirely understandable that they have adopted, as other researchers have reported (see McLaughlin *et al*. 2008) a highly medicalized understanding. The increasing availability of prenatal genetic tests raises concerns over the *message* that these tests convey about the value of life with a disability (Saxton 2000), and the *choices* of parents who decide not to terminate a pregnancy where an unborn child is identified as having a neurodevelopmental syndrome (Reinders 2000). But should these issues, which are tied to complex arguments over genetic testing and termination of pregnancies (*c.f*. Shakespeare 2013), be allowed to affect the health care received by people who have already been born? The minority of ‘professionals’, who expressed concerns about focusing on people's genetic syndromes, whether in the context of health care or social care, raised two related issues. Namely, that care and treatment should be person centred and that people should not be defined by their health risks. People with learning disabilities have, historically, been defined almost exclusively by their perceived deficiencies; deficits that, in the climate of the times, the medical profession catalogued, measured and used to justify incarceration in long‐stay hospitals (Rolph *et al*. 2005). There is justifiable concern, therefore, that a re‐medicalization of people's learning disabilities by focusing on the genetic causes of some people's disabilities could renew old practices and prejudices. But is this really possible, when the institutional basis for this, the long‐stay hospitals, are long gone, and national policies and legislation are clearly aimed at promoting equal rights, access and opportunities (see *Valuing People Now* 2009 and the *Care Act 2014*)? Moreover, how can people with disabilities, and in particularly those whose disabilities have a genetic origin, enjoy ‘the highest attainable standard of health’ (United Nations, 2006) if clinicians and DSS do not give due regard to their syndrome‐specific health risks? Surely, being cognizant of a person's genotype and health‐related risks is as much a part of providing person‐centred care and support, as respecting a person's will and preference? In other words, it might be time for an embodied understanding of people's impairments: one which recognizes the significance of both embodied health risks and the need for institutional reform to counter discrimination (Bill Hughes & Paterson 1997).

In sum, the case for a more judicious use of genetic knowledge in the design and delivery of healthcare and social care services for people whose learning disability has a genetic cause seems overwhelming. Introducing syndrome‐specific risks into annual health checks and care plans would involve negligible cost, while having the potential to save money where this resulted in the early detection and treatment of ill‐health. Multidisciplinary clinics convened two or three times a year would enable parents, and also social care providers, to access integrated services where a person's physical and mental health problems could be addressed by clinicians with the relevant expertise. Such clinics could also provide an opportunity for closer collaborations between research active clinicians, syndrome‐specific support groups and parents. Again, where these clinics enable the early detection of ill‐health, and lead to fruitful research partnerships, these might offset the necessary costs. With respect to raising awareness of syndrome‐specific health risk in both mainstream services (GP surgeries and general hospitals) and specialist community learning disability services, an NHS branded website that provided relevant information might be very useful. And again would not be particularly costly. The biggest barrier to any of these changes, however, is likely to be the small number of persons affected by any one syndrome. Although, with respect to the 11 syndromes discussed here, their combined prevalence is 479 per 250 000 of the population (see Table 2). The continued advance in genetic research, and the growing maturity of these syndrome support organisations, is likely to generate growing pressure for greater recognition of people's genetic syndromes in both health and social care services: biological citizenship. It may also be the case that the era when people with very different impairments mobilize under a shared experience of discrimination and oppression is coming to an end. We say this, because formal equality is now guaranteed under law, and the vast majority of people with impairments do not actually identify as *disabled* (Shakespeare 2013), while a significant proportion of people do seem willing to be identified through their medical diagnoses.

## Conclusion

The health and well‐being of those people whose learning disabilities are associated with conditions of genetic origin could be improved through a number of low‐tech reforms that give greater prominence to their syndrome‐specific health risks. Mental health and behavioural problems are more intractable; yet, research into behavioural phenotypes holds out the possibility of pharmacological and non‐pharmacological interventions that could enhance opportunities for participating in society. In summary, a medicalization of these populations’ learning disabilities would appear to be beneficial to the extent it is focused on interventions that potentially improve people's lives rather than catalogues their deficiencies. As such, the association at the centre of the social model of disability, which associates the medicalization of people's disabilities with social oppression, has to be seriously scrutinized. Otherwise, there is the distinct possibility that many people whose learning disabilities are associated with a condition of genetic origin will fail to benefit from advances in medical genetics.
